# Understanding and tackling snakebite envenoming with transdisciplinary research

**DOI:** 10.1371/journal.pntd.0010897

**Published:** 2022-11-17

**Authors:** José María Gutiérrez, Juliette Borri, Tamara Giles-Vernick, Romain Duda, Abdulrazaq G. Habib, Anita Malhotra, Gerardo Martín, Anna F. V. Pintor, Julien Potet, Terence Scott, Isabelle Bolon, Rafael Ruiz de Castañeda

**Affiliations:** 1 Instituto Clodomiro Picado, Facultad de Microbiología, Universidad de Costa Rica, San José Costa Rica; 2 Policy Cures Research, Sydney, Australia; 3 Anthropology & Ecology of Disease Emergence Unit, Institut Pasteur, Paris, France; 4 Infectious and Tropical Diseases Unit, Bayero University, Kano, Nigeria; 5 Molecular Ecology and Evolution at Bangor, School of Natural Sciences, College of Environmental Sciences and Engineering, Bangor University, Bangor, Gwynedd, United Kingdom; 6 Departamento de Sistemas y Procesos Naturales, Escuela Nacional de Estudios Superiores, Unidad Mérida, Universidad Nacional Autónoma de México, Yucatán, México; 7 Division of Data, Analytics and Delivery for Impact, World Health Organization, Geneva, Switzerland; 8 Australian Institute of Tropical Health and Medicine, Division of Tropical Health and Medicine, James Cook University, Cairns, Australia; 9 Médecins Sans Frontières Access Campaign, Geneva, Switzerland; 10 Global Alliance for Rabies Control, Manhattan, Kansas, United States of America; 11 Institute of Global Health, Department of Community Health and Medicine, Faculty of Medicine, University of Geneva, Geneva, Switzerland; Fundação de Medicina Tropical Doutor Heitor Vieira Dourado, BRAZIL

Snakebite envenoming (SBE) is a neglected tropical disease (NTD) of high global impact. The World Health Organization (WHO) estimates 4.5 to 5.4 million people are bitten by snakes annually, resulting in 1.8 to 2.7 million envenomings, 81,000 to 138,000 deaths, and at least 400,000 people suffering from physical or psychological sequelae [1,2]. SBE mostly affects impoverished rural populations in sub-Saharan Africa, Asia, Latin America, and parts of Oceania, thus fueling a vicious cycle of poverty and illness. SBE not only affects humans, but also domestic animals, including livestock, with negative social and economic consequences [3,4]. This requires a better understanding of the complex social, cultural, and ecological contexts where SBE occurs [5], within the conceptual frame of One Health, an integrated approach that recognizes the health of humans, animals, and the environment as closely linked and interdependent [6]. Such complexity demands more integrative approaches for better understanding and confronting this disease.

SBE has unique features that make its prevention and control challenging. Unlike many infectious diseases, SBE cannot be eradicated, but its incidence and impact can be reduced through effective programs aimed at better prevention and rapid access to treatment. This in turn demands the engagement of communities to improve the cohabitation of humans, domestic animals, and snakes in rural agroecosystems. In 2019, the WHO launched a strategy for the prevention and control of SBE, aimed at halving the deaths and disabilities caused by this NTD by the year 2030. This strategy is based on 4 pillars, i.e., empower and engage communities; ensure safe, effective treatment; strengthen health systems; and increase partnerships, coordination, and resources [2]. Building on previous ideas and publications, this article discusses and advocates for transdisciplinary research on SBE and for promoting dialogue and collaboration between sectors, particularly by engaging communities affected by SBE at all levels of the research process.

## Towards transdisciplinary research in SBE

Notable research efforts have been made over decades to build a body of knowledge in areas dealing with SBE, such as herpetology, toxinology, immunology, epidemiology, clinical sciences, and public health, among others. As part of these efforts, several research agendas have been proposed, involving basic studies on venom composition and mechanisms of action; preclinical assessment of the efficacy of antivenoms and new therapies; epidemiology and clinical research of SBE, including diagnosis and treatments; technological development of antivenoms and novel therapies; and public health intervention policies, among other topics [7,8]. Such high-quality research is essential to provide further understanding upon which to build solid knowledge-based interventions in the public health realm.

In addition to these ongoing specialized efforts, there is an urgent need to expand the research landscape in the field of SBE by the incorporation of a transdisciplinary perspective. According to Jahn and colleagues [9], **“**Transdisciplinarity is a reflexive research approach that addresses societal problems by means of interdisciplinary collaboration as well as the collaboration between researchers and extra-scientific actors; its aim is to enable mutual learning processes between science and society; integration is the main cognitive challenge of the research process.” The need for transdisciplinarity is inherent to the 4 pillars upon which the WHO SBE strategy is based [2]. Recently, a collection of articles was published underscoring the value of the transdisciplinary perspective in the field of SBE [10]. Moreover, renewed interdisciplinary efforts are gaining momentum in the SBE field (see for example [11]).

Transdisciplinary projects should include 3 key elements [9]: (a) Facilitate the integration between different disciplinary fields, not just to juxtapose them. This implies the need to think outside “disciplinary boxes,” not only for finding areas of overlap, but also to transgress the limits between disciplines in a creative process of mutual learning. (b) Incorporate diverse social sectors and stakeholders in the design, development, analysis, and translation of the results of the project. (c) Apply a translational approach, i.e., that the research outcomes should be applied in the solution of the problem through effective interventions. Thus, integration is not only relevant to scientific disciplines, but also between scientists and other social actors. This demands rejecting the traditional power asymmetries in research between academic and non-academic actors. Overall, the main challenge of transdisciplinarity is the real integration between disciplines and between scientists and other members of the society, as well as the effective translation of the research findings to solve a societal problem while promoting capacity building at the local level.

## How to develop transdisciplinary research in SBE?

The development of transdisciplinarity in the field of SBE demands innovative approaches. Lessons should be learned from ongoing efforts on transdisciplinarity in various disciplines, and valuable methodological toolboxes for transdisciplinary research are available [12,13]. These experiences and tools place a strong emphasis on the integration of a diverse set of scientific fields with the knowledge generated by the local communities in a genuine “dialogue of knowledges.” On these bases, we suggest a general approach based on understanding the local context and promoting scientific and non-scientific actor dynamics and integration as guiding principles for the design and development of transdisciplinary research projects. Firstly, research should be rooted in specific contexts in localities where snake bites are prevalent, involving various levels in the identification of the settings, e.g., local community, state/province, or country. This demands, at an initial stage, the building up of a large cross-sectorial dataset on the features of the setting, as they relate to SBE, using many sources of information and involving diverse actors in local communities, i.e., organized community groups, local leaders, regional leaders, people affected by SBE (farmers, herdsmen, children), with a gender focus to ensure participation of women and girls, traditional healers, and representatives of the local and national governments, among others. Thus, since the first stages in the research process, i.e., the identification of the problem to be studied, the relevant stakeholders to be involved, as well as the design of the research itself, the local communities should have a protagonist role.

In the research design process, the next step should start through a dynamic, iterative exchange of ideas between specialists on various scientific disciplines and stakeholders of the society where the study will be developed. Research teams should be not only cross-sectorial and interdisciplinary, but also truly international, involving local experts and non-scientific actors, and should be balanced from a gender perspective. The aim of this process is to identify the main research questions and the prioritization of the main needs/gaps to be addressed. From academia, the emphasis needs to be placed on integrating researchers from as many disciplines as required, depending on the knowledge gaps and research issues identified, in partnership with local communities and based on the evolution of the project. Scientists and local community participants should work in a highly integrated fashion instead of in silos. This open communication and exchange between researchers and non-scientific actors must be sustained throughout the project to respond to challenges and obstacles, having a broader impact on the local cultural, educational, and public health landscape.

Rooted in local contexts, this integrative scheme is a space co-creation from which the project design, implementation, and operationalization emerges and adapts to local priorities and conditions. This demands a dynamic and permanent exchange of views and analyses throughout the research and implementation process. This in turn will generate integrative knowledge on the various aspects of the problem while simultaneously facilitating plans to translate research findings into implementation of actions that will improve the prevention and management of SBE at various levels depending on the project (local, regional, national).

## Concluding remarks

**Fig 1:**
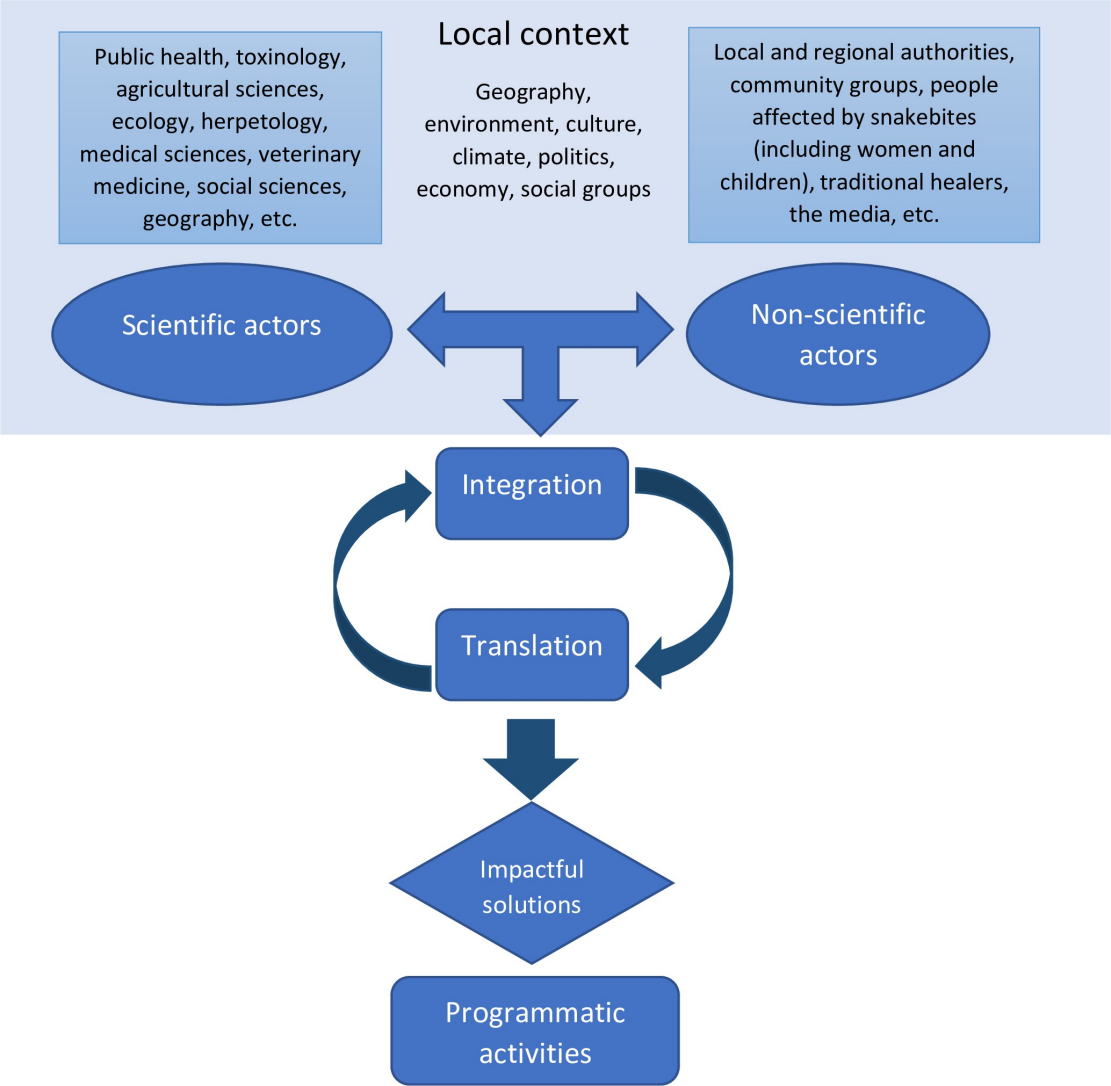
Flowchart summarizing the transdisciplinary research approach proposed for snakebite envenoming. The key concept is integration at all levels, as an inclusive dynamic and iterative process between and within the 3 pillars (local context, scientific disciplines, and non-scientific actors). Such integrative and participatory perspective should be present from the design of the project up to its completion. Through the integration of the research process between the pillars, translating research outcomes from project to policy and reality can be done more seamlessly resulting in impactful solutions aimed at reducing the impact of snakebite envenoming.

This innovative and participatory approach to designing and implementing research in the field of SBE aims to overcome the main cognitive challenge of research: integration at many levels. Using this transdisciplinary scheme, projects will be integrated into the local context, academic and non-academic actors will be involved in the co-design and execution of research, and the output will be inherently integrated in the fabric of science and society as a consequence of where it was undertaken. Translating research outcomes into thoughtful, evidence-based solutions implemented beyond the research project setting is only feasible because of the involvement of affected people as well as wider governing bodies that provide resources and support to implement the solution, as shown in Fig 1. Renewed work is needed in this area to explore the possibilities and challenges of this approach, which could be also applied to other diseases. Moreover, it is necessary to scale up these local initiatives to extend its impact more broadly and to promote sustainability. As the research, policy, and funding landscape for SBE evolves in the next decade, we need to prioritize translation and integration, within the frame of One Health and transdisciplinarity, as key mechanisms to ensure impact and success in managing this NTD.

## References

[1] Gutiérrez JM, Calvete JJ, Habib AG, Harrison RA, Williams DJ, Warrell DA. Snakebite envenoming. Nat Rev Dis Primers. 2017;3(2017):79. doi: 10.1038/nrdp.2017.79 28980622

[2] World Health Organization. Snakebite Envenoming. A Strategy for Prevention and Control. World Health Organization, Geneva. 2019. Available from: https://apps.who.int/iris/bitstream/handle/10665/324838/9789241515641-eng.pdf.

[3] Bolon I, Finat M, Herrera M, Nickerson A, Grace D, Schutte S, et al. Snakebite in domestic animals: First global scoping review. Prev Vet Med. 2019;170:104729. doi: 10.1016/j.prevetmed.2019.104729 31421490

[4] Babo Martins S, Bolon I, Alcoba G, Ochoa C, Torgerson P, Sharma SK, et al. Assessment of the effect of snakebite on health and socioeconomic factors using a One Health perspective in the Terai region of Nepal: a cross-sectional study. Lancet Glob Health. 2022;10:e409–e415. doi: 10.1016/S2214-109X(21)00549-0 35180422

[5] Duda R, Monteiro WM, Giles-Vernick T. Integrating lay knowledge and practice into snakebite prevention and care in central Africa, a hotspot for envenomation. Toxicon:X. 2021;11:100077. doi: 10.1016/j.toxcx.2021.100077 34381993 PMC8334740

[6] One Health High Level Expert Panel (OHHLEP), Adisasmito WB, Almuhairi S, Behravesh CV, Bilivogi P, Bukachi SA, et al. One Health: A new definition for a sustainable and healthy future. PLoS Pathog. 2022;18:e1010537. doi: 10.1371/journal.ppat.1010537 35737670 PMC9223325

[7] Gutiérrez JM, Theakston RDG, Warrell DA. Confronting the neglected problem of snake bite envenoming: the need for a global partnership. PLoS Med. 2006;3:e150. doi: 10.1371/journal.pmed.0030150 16729843 PMC1472552

[8] Harrison RA, Gutiérrez JM. Priority actions and progress to substantially and sustainably reduce the mortality, morbidity and socioeconomic burden of tropical snakebite. Toxins (Basel). 2016;8:351. doi: 10.3390/toxins8120351 27886134 PMC5198546

[9] Jahn T, Bergmann M, Keil F. Transdisciplinarity: Between mainstreaming and marginalization. Ecol Econ. 2012;79:1–10. doi: 10.1016/j.ecolecon.2012.04.0178

[10] Gutiérrez JM, Bolon I, Ruiz de Castañeda R, editors. Special issue: A transdisciplinary view of snakebite envenoming. Toxicon:X. 2022. Available from: https://www.sciencedirect.com/journal/toxicon-x/special-issue/10H1829SGCF. 10.1016/j.toxcx.2021.100088 PMC8718666 35005608

[11] Alcoba G, Ochoa C, Babo Martins S, Ruiz de Castañeda R, Bolon I, Wanda F, et al. Novel transdisciplinary methodology for cross-sections analysis of snakebite epidemiology at national scale. PLoS Negl Trop Dis. 2021;15:e0009023. doi: 10.1371/journal.pntd.0009023 33577579 PMC7906452

[12] Swiss Academy of Sciences. Methods and tools for co-producing knowledge. Available from: https://naturalsciences.ch/co-producing-knowledge-explained/methods/td-net_toolbox.

[13] Scheling E, Zinsstag J. Transdisciplinary research and One Health. In One Health. The theory and practice of integrated health approaches. Zinsstag J, Schelling E, Waltner-Toews D, Whitaker M, Tanner M, editors. Oxfordshire UK: CABI; 2015. p. 366–373.

